# Relationships of growth factors, proinflammatory cytokines, and anti-inflammatory cytokines with long-term clinical results of autologous bone marrow mononuclear cell transplantation in STEMI

**DOI:** 10.1371/journal.pone.0176900

**Published:** 2017-05-30

**Authors:** Vyacheslav V. Ryabov, Marina A. Kirgizova, Tatiana E. Suslova, Sergei I. Karas, Valentin A. Markov, Rostislav S. Karpov

**Affiliations:** 1 Cardiology Research Institute, Tomsk National Research Medical Center, Tomsk, Russian Federation; 2 Siberian State Medical University, Department of Cardiology at the Faculty of Continuous Medical Education, Tomsk, Russian Federation; 3 National Research Tomsk State University, Laboratory of Translational Cellular and Molecular Biomedicine, Tomsk, Russian Federation; Universita degli Studi di Parma, ITALY

## Abstract

**Aim:**

The aim of the study was to test the hypothesis suggesting that the pre-intervention levels of proinflammatory cytokines, anti-inflammatory cytokines, and angiogenic growth factors predict the long-term clinical results of autologous bone marrow-derived mononuclear cell (ABMMC) transplantation in patients with primary ST elevation myocardial infarction (STEMI).

**Methods and results:**

From 2003 to 2006, a total of 62 patients with primary STEMI were enrolled in an open randomized study registered under the title ESTABOMA. Patients were randomized into two groups: group 1 included patients treated with percutaneous coronary intervention (PCI) and ABMMC transplantation (n = 28); group 2 comprised patients treated only with PCI (n = 34). Follow-up study was performed 7.96 ± 0.96 years after STEMI and involved physical examination, six-minute walk test, echocardiography, and determination of brain natriuretic peptide (BNP) levels. The total and cardiovascular mortality rates were higher in group 1 compared with group 2: 36% (n = 10) vs. 12% (n = 4) (p = 0.02) and 29% (n = 8) vs. 6% (n = 2) (p = 0.03), respectively. Lower levels of proinflammatory cytokines were observed in group 1 after PCI and ABMMC transplantation. Serum levels of FGF, VEGF, and IL-10, determined before PCI and ABMMC transplantation were prognostically significant long-term indicators of unfavorable course of CAD after STEMI.

## Introduction

Significant progress has been achieved in the treatment of ST elevation myocardial infarction (STEMI) based on timely reperfusion therapy and early percutaneous coronary intervention (PCI) that improve short-term and long-term outcomes. However, acute myocardial infarction (AMI) remains a leading cause of death and chronic heart failure (CHF). Effective reperfusion of infarct-related artery allows to limit infarct size. However, aseptic inflammation develops in the myocardium in response to ischemic injury. This further leads to left ventricular dysfunction and progressive ventricular remodeling.

Several clinical trials addressing autologous bone marrow mononuclear cell (ABMMC) transplantation have been performed in STEMI patients. The main purpose of ABMMC transplantation is to stimulate myocardial recovery through the stimulation of scar formation, cardiomyocyte regeneration, and neoangiogenesis. According to data available in MEDLINE, EMBASE, CENTRAL, CINAHL, and ClinicalTrials.gov, over 30 placebo-controlled trials were conducted to determine the efficacy and the safety of ABMMC transplantation [1, 2]. The initial short-term and long-term results of the randomized trials were overoptimistic whereas further studies generated modest clinical results [2, 3, 4]. In recent years, several experimental studies investigated the effects of angiogenic growth factors on the development of collateral blood vessels in ischemic tissue and on the regeneration in the infarct zone [5–9]. Evidence suggests that immunoinflammatory processes are upregulated in patients with AMI; an imbalance between proinflammatory and anti-inflammatory cytokines may adversely affect the healing process in the area of ischemic damage increasing the risk of complications [10]. However, the results of clinical trials still remain limited. It has been recognized that one of the main causes underlying poor clinical efficacy of ABMMC transplantation is associated with low retention, survival, and viability of engrafted cells in the area of transplantation. Experimental models show that inflammatory reactions control regeneration after the ischemic injury and influence the abilities of progenitor cells to differentiate into cardiomyocytes, endotheliocytes, or into inflammatory cells (macrophages) [11, 12]. Moreover, active inflammation stimulates apoptosis and necrosis of transplanted cells [11]. It remains unclear how the angiogenic growth factors, proinflammatory cytokines, and anti-inflammatory cytokines affect the results of cell therapy in STEMI patients. Associations of the serum levels of angiogenic growth factors and cytokines with the long-term efficacy of ABMMC transplantation in STEMI patients also remain unknown and require further investigation. Earlier, we reported the results of an open randomized study registered under the title ESTABOMA [13–15]. Here we present the long-term results of ABMMC transplantation in patients with primary STEMI with regard to the baseline serum levels of growth factors, proinflammatory cytokines, and anti-inflammatory cytokines.

The aim of the study was to test the hypothesis suggesting that the pre-intervention levels of proinflammatory cytokines, anti-inflammatory cytokines, and angiogenic growth factors predict the long-term clinical results of ABMMC transplantation in patients with primary STEMI.

## Materials and methods

### Study design and population

A total of 62 primary STEMI patients, admitted to Cardiology Research Institute of Tomsk NRMC (Tomsk, Russian Federation) from December 1, 2003 to December 20, 2006, were enrolled in an open randomized study registered under the title ESTABOMA at http://www.clinicaltrials.gov (trial number NCT01748383). Inclusion criteria were age 18 to 75 years, primary STEMI, reperfusion time of the infarct-related coronary artery (IRCA) longer than 4 h after primary STEMI onset, and admission to Coronary Care Unit within 24 hours after beginning of STEMI. Exclusion criteria were permanent form of atrial fibrillation, any valvular heart disease, severe comorbidity, and patient refusal to undergo the necessary studies.

**Fig 1:**
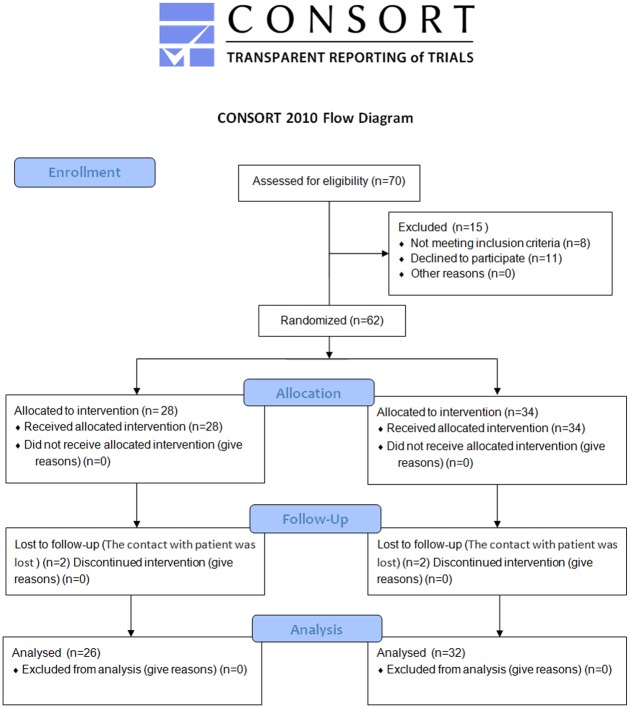
CONSORT flow diagram.

In our clinic, randomization was achieved using the method of opaque sealed envelopes each of which contained information on what group a patient is assigned to: to ABMMC transplantation group (group 1) or to control group (group 2). According to the protocol, a clinical investigator assigned one envelope to a patient during the enrolment into the study; after that, patient unsealed the envelope to find out which group he or she was randomized into. After that, the envelope was handed back to clinical investigator. Each patient had a 50–50 chance to be assigned to one or another group. Group 1 comprised patients who underwent PCI and ABMMC transplantation at day 20 ± 10 after STEMI onset (n = 28); group 2 comprised patients who underwent only PCI (n = 34). The study design is shown in Fig 1.

Baseline patient characteristics (n = 62) are presented in Table 1 showing that the groups were comparable.

**Table 1:** Baseline patient characteristics.

Characteristic	Group 1M ± SD, n (%)	Group 2M ± SD, n (%)	p
Number of pts	28	34	
Mean age	55.3 ± 8.2	52.8 ± 8.5	0.29
Male/female	25 (89%)/3 (11%)	27 (79%)/7 (21%)	0.26/0.07
Smoking	23 (82%)	26 (76%)	0.61
Obesity	9 (32%)	12 (35%)	0.37
Hypertension	21 (75%)	30 (88%)	0.18
Preinfarction angina	13 (46%)	18 (53%)	0.81
Anterior myocardial infarction	23 (82%)	24 (71%)	0.35
IRCA ADA / RCA / CA	22 (79%)/4 (14%)/2 (7%)	24 (71%)/7 (21%)/3 (8%)	0.21/0.16/0.31
Coronary artery lesion 1- / 2- / 3-vessel disease	3 (11%)/18 (64%)/ 6 (21%)	13 (38%)/15 (44%)/6 (18%)	0.36/0.41/0.28
Reperfusion time of IRCA, h	5.7 ± 0.5	6.5 ±1.4	0.10
Acute heart failure, functional class I/II/III/IV	11 (39%)/11 (39%)/4 (14%)/2 (7%)	15 (44%)/14 (42%)/5 (15%)/-	0.40
Complete revascularization	12 (43%)	17 (50%)	0.57

Note: ABMMC: autologous bone marrow mononuclear cells; ADA: anterior descending artery; CA: circumflex artery; IRCA: infarct-related coronary artery; RCA: right coronary artery; pts: patients.

A total of 100 mL bone marrow was aspirated from the patient's wing of the ilium. Then, ABMMC were isolated by the method of gradient centrifugation (density gradient Histopaque-1077). Cell viability rates were estimated based on vital staining with trypan blue and ranged from 98% to 99%. Obtained ABMMC contained CD34+ cells up to 2.4 ± 0.64 × 10^5^ (CI 2.2–3.8) per mL and CD34+CD38– bone marrow cells up to 5.3±1.7 × 10^4^ (CI 3.9–6.5) per mL (FACSCalibur, Biosciences, USA). ABMMC were introduced into the IRCA lumen by the method of passive seeding [7,8]. Distribution of ^99m^Tc-HMPAO-labeled mononuclear cells in the patient’s body was studied by scintigraphy (Nuclear Gamma Camera 500, Technicare, USA-Germany). The study protocol is presented at http://www.clinicaltrials.gov and is described in previously published articles [13, 15].

### Measurements of proinflammatory cytokines, anti-inflammatory cytokines, and growth factors

Plasma samples were collected to determine the serum levels of cytokines including tumor necrosis factor α (TNFα), interleukin (IL)-1β, IL-2, IL-4, IL-6, IL-8, IL-10 and growth factors including hepatocyte growth factor (HGF), vascular endothelial growth factor (VEGF), fibroblast growth factors (FGF), and insulin-like growth factors (IGF) before PCI and 2, 5, and 12 days after PCI in patients of both groups. The levels of cytokines and growth factors were assessed by enzyme-linked immunosorbent assay as follows: HGF, FGF (Biosource, Belgium), VEGF (Cytimmune, USA), IGF (DSL, USA), IL-1β, IL-2, IL-4, IL-6, IL-8, and IL-10 (Protein Contour, Russia).

### Follow-up examination and outcomes

A follow-up study was performed 7.96 ± 0.96 years (CI 7.70–8.22) after STEMI and involved physical examination, six-minute walk test (6MWT), echocardiography (VIVID 7, GE), and determination of serum brain natriuretic peptide (BNP) (Triage Meter, Biosite, USA). Based on echocardiography data, we assessed mean changes from baseline in the left ventricular end-diastolic volume (LVEDV), left ventricular end-systolic volume (LVESV), left ventricular end-diastolic index (LVEDI), left ventricular end-systolic index (LVESI), left ventricular ejection fraction (LVEF), and wall motion score index (WMSI). Left ventricular ejection fraction was calculated by Simpson's rule.

Death, repeated myocardial infarction (RMI), unstable angina, chronic heart failure (CHF) NYHA class ≥ II, and stroke were primary endpoints.

Safety endpoints of ABMMC transplantation were clinically significant cardiac arrhythmias including life-threatening arrhythmias and newly diagnosed oncological diseases.

Information on the vital status and on the clinical course of cardiovascular disease was obtained from the medical documentation (clinical records and patients' cards), telephone interviews, and from the questionnaires. In case of death, information was acquired from the death certificates and from the postmortem study protocols.

### Ethics

The protocol of the study was approved by the local Committee on Biomedical Ethics of Cardiology Research Institute, Tomsk NRMC (Tomsk, Russian Federation): protocol #27 from November 22, 2003. The study complied with the Declaration of Helsinki Guidelines. Written informed consent was obtained from all patients.

### Statistical analyses

The study was designed as a pilot study. Considering that the initial distribution of patient characteristics was unknown, it was impossible to calculate the required sample size. Quantitative data are presented as mean (M) and standard deviation (SD); mean (M) and confidence interval (CI); and median (Me) and interquartile range (Q25 –Q75). Distribution of variables was estimated by the Kolmogorov—Smirnov test with the Lilliefors correction, Shapiro—Wilk test, and by the method of histogram visualization. Homogeneity of variances was assessed by the Levene's test. For quantitative parameters, the Mann-Whitney test was used to compare differences between two independent groups; the Friedman test and Bonferroni correction were used to compare differences between three and more dependent groups; and the Wilcoxon signed rank test was used for pair-wise comparison of two dependent groups. Multiple comparisons were performed by the Dunn's test. Contingency tables were analyzed for statistical processing of qualitative data: Pierson χ^2^ test was used for independent groups; two-sided exact Fisher's test and χ^2^ test with Yates' correction were used for tables with expected cell frequencies less than 5 (for 2×2 contingency tables). To study relationships between the variables, correlation analysis with the Spearman, Kendall and Gamma rank correlation coefficients was performed. Survival curves were built by the Kaplan-Meyer method; comparison between curves was performed by the Cox test. In our study, vast majority of our variables were not normally distributed. That was the reason why we have chosen to use the simple method of logistic regression. To identify predictors of adverse endpoints, logistic regression method and ROC curve analysis were used. Values were considered statistically significant when P was < 0.05.

Statistical processing of data was done by using the following software: SAS 8 (USA), SPSS Statistic 19 (USA), R (USA), and Statistica 10.0 for Windows (Stat Soft, Inc., USA).

### Mathematical analysis

Mathematical analysis was conducted to assess the prognostic values of growth factors and cytokines in relation to the long-term survival of patients. To achieve this, multi-dimensional mathematical model was created based on the k-Nearest Neighbors algorithm; vital status at a time of the control examination was selected as a grouping variable. We used algorithms Add (sequential addition of signs) and Del (successive elimination of signs) for the selection of the most informative properties. All algorithms were implemented in MathCad 14 (USA, 2009).

## Results

**Fig 2:**
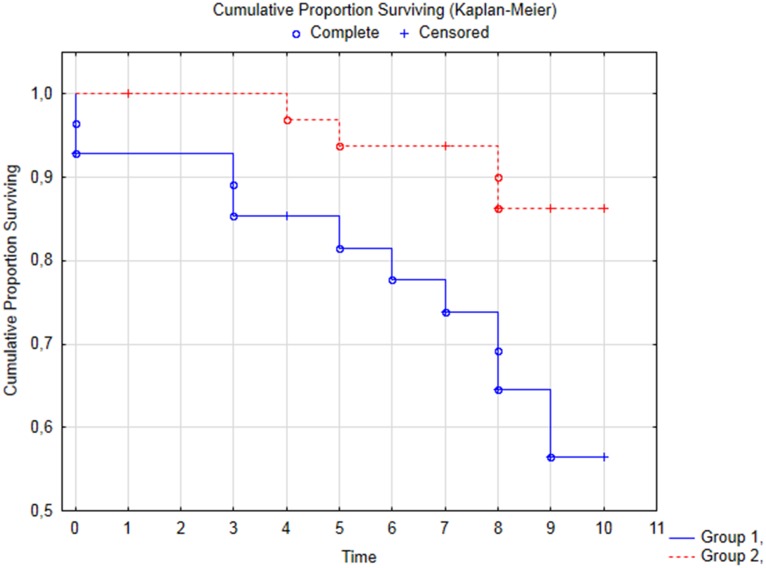
Kaplan-Meier survival estimates for cause of death (Cox's F test p = 0.01). Note: Fig 2 shows that proportion of survived patients at the moment of control study was lower in group 1.

Vital status information was obtained from 58 patients (93%). Forty-four patients (70%) underwent a follow-up examination. During a median follow-up period of 7.96 ± 0.96 years (CI 7.70–8.22), 14 patients (22%) died. Total incidence of deaths in group 1 was higher compared with that in group 2: 10 pts (36%) vs. 4 pts (12%), p = 0.02, respectively (Fig 2).

The rates of RMI, stroke, repeated PCI, repeated coronary artery bypass grafting (CABG), and angina functional class 2 and higher did not significantly differ between groups. However, the rate of hospitalizations due to unstable angina was significantly higher in control group compared with ABMMC transplantation group: 35% (12) vs. 18% (5), p = 0.05.

Chronic heart failure of class II and higher was found more often in group 2: 4 cases (14%) vs. 12 cases (35%), p = 0.05. However, exercise tolerance (6MWT: 499.11 ± 92.53 m vs. 488.19 ± 189.84 m, p = 0.42) and BNP levels (M [CI]: 148 pg/mL [62.24–235.72] vs. 169 pg/mL [51.26–308.13],p = 0.34) did not significantly differ between group 1 and group 2.

No differences in safety endpoints were found (Table 2). During the entire period of the follow-up study, the frequencies of clinically significant cardiac arrhythmias were similar in both groups (14% vs. 9%, p = 0.42); no life-threatening arrhythmias were documented. The frequencies of newly diagnosed oncological diseases did not differ between the groups (4% vs. 6%, p = 0.79) (Table 2).

**Table 2:** Outcomes and safety.

Outcome events	Group 1 (n = 28), n (%)	Group 2 (n = 34), n (%)	p
Cardiovascular death	8 (29%)	2 (6%)	0.03
Non-cardiovascular death	1 (4%)	2 (6%)	0.69
The cause of death is unknown	1 (4%)	-	-
Total incidence of deaths	10 (36%)	4 (12%)	0.02
Hospitalizations due to unstable angina	5 (18%)	12 (35%)	0.05
Repeated myocardial infarction	6 (21%)	5 (14%)	0.62
Stroke	1 (4%)	3 (9%)	0.64
Chronic heart failure class ≥ II	4 (14%)	12 (35%)	0.05
Stable angina class ≥ II	1 (4%)	5 (14%)	0.14
Repeated PCI	3 (11%)	3 (9%)	0.53
Aneurysmectomy	1 (4%)	-	
CABG	1 (4%)	2 (6%)	0.63
**Safety endpoints**			
New arrhythmias	4 (14%)	3 (9%)	0.42
Oncology diseases	1 (4%)	2 (6%)	0.79

Moreover, analysis of echocardiographic parameters did not show significant differences between the groups (Table 3).

**Table 3:** Echocardiographic parameters.

Echocardiographic parameters	Group 1 (n = 16), M [CI]	Group 2 (n = 28), M [CI]	p
Left ventricular end-diastolic volume, mL			
Baseline	119.05 [110.25–127.84]	106.31 [86.20–113.88]	0.12
After 7.96±0.96 years	138.37 [120.86–155.88]	120.30 [104.14–138.21]	0.42
Left ventricular end-systolic volume, mL			
Baseline	61.35 [55.45–67.25]	51.11 [38.34–57.65]	0.12
After 7.96±0.96 years	75.47 [60.83–90.11]	56.73 [43.50–64.21]	0.36
Left ventricular end-diastolic index, mL/m^2^			
Baseline	60.41 [49.95–85.21]	51.90 [66.04–45.21]	0.89
After 7.96±0.96 years	69.90 [61.31–78.50]	69.44 [46.94–51.21]**	0.32
Left ventricular end-systolic index, mL/m^2^			
Baseline	37.91 [30.01–44.44]	35.50 [28.22–45.21]	0.09
After 7.96±0.96 years	44.91 [36.94–51.21]*	31.71 [23.67–43.15]*	0.41
Left ventricular ejection fraction, %			
Baseline	49.07 [46.94–51.21]	50.85 [45.94–56.23]	0.29
After 7.96±0.96 years	48.60 [46.01–52.18]	54.35 [46.94–51.21]	0.51
Wall motion score index			
Baseline	1.71 [1.54–1.88]	1.74 [1.64–1.90]	0.84
After 7.96±0.96 years	1.55 [1.43–1.66]	1.52 [1.41–1.63]***	0.68

*—р ≤ 0.05 for intragroup comparisons of dynamics of left ventricular end-systolic index in group 1 and 2;

**—р ≤ 0.05 for intragroup comparisons of dynamics of left ventricular e end-diastolic index in group 2;

***—р ≤ 0.05 for intragroup comparisons of dynamics of wall motion score index in group 2.

At the next stage of the study, we determined the levels of proinflammatory cytokines, anti-inflammatory cytokines, and angiogenic growth factors in patients with STEMI at different time points. The levels of growth factors and cytokines are presented in Table 4.

**Table 4:** Serum levels of growth factors and cytokines.

Growth factors and cytokines	Day	Group 1 (n = 21), M [CI]	Group 2 (n = 30), M [CI]	p
HGF, pg/mL	baseline	914.01 [548.37–1279.64]	837.12 [639.67–1034.54]	0.88
	day 2	1019.05 [814.71–1409.76]	967.13 [876.90–1254.43]	0.56
	day 5	1997.36 [1440.87–2553.85]*	1817.66 [1290.76–2344.57] *	0.68
	day 12	549.15 [422.01–676.29]**	595.26 [460.25–730.26] **	0.74
VEGF, pg/mL	baseline	201.18 [96.93–305.43]	229.78 [150.87–308.69]	0.46
	day 2	202.01 [28.82–375.21]	244.84 [57.38–432.31]	0.93
	day 5	113.75 [68.66–158.84]	170.18 [105.7–234.67]	0.37
	day 12	149.71 [91.23–208.18]	157.28 [110.04–2045.32]	0.57
FGF, pg/mL	baseline	15.75 [13.12–18.39]	16.95 [14.27–19.64]	0.80
	day 2	17.75 [9.54–25.97]	19.21 [13.11–25.3]	0.75
	day 5	16.96 [14.35–19.62]	16.31 [13.32–19.32]	0.61
	day 12	14.82 [12.67–16.94]	15.15 [12.23–18.06]	0.64
IGF, mcg/mL	baseline	185.88 [164.27–207.53	186.11 [163.17–209.05]	0.70
	day 2	171.11[105.12–236.88]	177.12 [144.36–209.89]	0.63
	day 5	168.54 [147.33–189.76]	167.22 [144.51–189.93]	0.80
	day 12	183.07 [165.76–200.37]	175.62 [152.62–198.62]	0.46
TNFα, pg/mL	baseline	47.71 [18.86–76.53]	46.15 [22.74–69.55]	0.77
	day 2	40.92 [14.55–67.29]	76.93 [27.63–126.24]	0.10
	day 5	45.75 [16.22–75.28]	119.78 [58.55–181.01]	**0.02**
	day 12	40.69 [10.65–70.72]	110.23 [41.84–178.62]	**0.05**
IL-1β, pmol/mL	baseline	86.65 [18.81–154.5]	150.27 [77.83–222.74]	0.25
	day 2	42.64 [12.01–73.27]	138.46 [69.48–207.44]	**0.01**
	day 5	75.04 [20.74–129.34]	136.35 [73.93–198.81]	0.08
	day 12	69.63 [22.53–116.72]	108.42 [56.44–160.39]	0.06
IL-2, pmol/mL	baseline	15.36 [9.61–21.12]	15.22 [10.63–19.81]	0.93
	day 2	12.92 [8.91–16.95]	13.12 [8.41–17.83]	0.96
	day 5	13.43 [8.87–17.98]	15.75 [10.69–20.82]	0.48
	day 12	13.12 [8.46–17.78]	15.34 [9.41–20.59]	0.65
IL-4, pmol/mL	baseline	90.98 [45.13–136.87]	144.59 [84.02–205.15]	0.24
	day 2	68.08 [23.38–112.77]	124.39 [74.45–174.33]	**0.02**
	day 5	64.05 [31.63–96.46]	136.57 [86.71–186.44]	**0.01**
	day 12	54.61 [26.65–82.57]	116.24 [56.24–176.24]	0.08
IL-6, pmol/mL	baseline	3.94 [2.79–5.08]	4.23 [3.25–5.21]	0.65
	day 2	5.63 [3.46–7.81]	5.75 [3.96–7.55]	0.96
	day 5	5.05 [3.31–6.81]	4.72 [2.58–6.86]	0.52
	day 12	3.79 [2.97–4.63]	5.17 [3.21–7.14]	0.86
IL-8, pmol/mL	baseline	55.71 [19.24–92.18]	79.04 [40.23–117.88]	0.18
	day 2	31.98 [11.54–52.43]	57.24[25.38–89.02]	0.14
	day 5	29.29 [9.02–49.55]	50.87 [28.53–73.21]	**0.005**
	day 12	50.31 [16.87–83.75]	52.32 [28.16–76.48]	0.26
IL-10, pmol/mL	baseline	3.02 [2.47–3.57]	3.09 [2.46–3.73]	0.81
	day 2	3.14 [2.63–3.64]	3.66 [2.37–4.95]	0.61
	day 5	4.17 [3.28–5.05]	3.41 [2.66–4.16]	0.32
	day 12	3.39 [2.87–3.91]	3.29 [2.65–3.93]	0.64

*—р ≤ 0.05 for intragroup comparisons of dynamics of serum levels in HGF baseline to day 5 in group 1 and 2;

**—р ≤ 0.05 for intragroup comparisons of dynamics of serum levels in HGF for days 5–12.

We found bidirectional trends in the changes of angiogenic growth factors. However, neither their absolute values nor their changes significantly differed between the groups.

Statistically significant differences between the groups were found in the IL-1β and IL-4 levels at day 2, in the TNFα, IL-4, and IL-8 levels at day 5, and in the TNFα levels at day 12 after PCI (p < 0.05). Lower levels of proinflammatory cytokines were observed in group 1 after PCI and ABMMC transplantation. In addition, the concentrations of anti-inflammatory cytokine IL-4 were lower at days 2 and 5 in group 1 after PCI and ABMMC transplantation (68.08 vs. 124.39 pmol/mL and 64.05 vs. 136.57 pmol/mL, respectively).

Dynamic changes in concentrations of cytokines and growth factors between time points were studied and the results are presented in Table 5.

**Table 5:** The percentage changes in the serum levels of growth factors and cytokines.

Changes in serum levels of growth factors and cytokines	Time period	Group 1 (n = 21), Me (Q25-Q75)	Group 2 (n = 30), Me (Q25-Q75)	p
% Δ HGF	Between baseline and day 2	24.01 (-9.03; 47.30)	21.63 (-13.27; 49.15)	0.12
	Between day 2 and day 5	13.51 (-1.08; 17.67)	9.13 (-8.77; 28.54)	0.68
	Between baseline and day 5	35.59 (-45.58; 307.96)^£^	19.95 (-2.73; 272.58) ^£^	0.78
	Between day 5 and day 12	-63.14 (-79.89; -16.79)	-57.81 (-85.27; -36.54)	0.46
	Between baseline and day 12	-7.71 (-53.49; 6.03)^γ^	-36.45 (-48.63; -20.13) ^γ^	0.24
% Δ VEGF	Between baseline and day 2	-40.01 (-90.03; 19.30)	-21.01 (-63.27; 13.95)	0.91
	Between day 2 and day 5	-51.76 (-82.66; 16.21)	-18.68 (-72.72; 121.93)	0.58
	Between baseline and day 5	-64.82 (-93.04; -0.99)	-42.55 (-81.56; 4.97)	0.41
	Between day 5 and day 12	-14.31 (-61.74; 21.64)	-0.49 (-39.85; 53.04)	0.32
	Between day baseline and day 12	-49.46 (-83.72; 24.54)	-50.87 (-65.95; -7.82)	0.20
% Δ FGF	Between baseline and day 2	-6.57 (-21.01; 78.58)	-8.62 (-17.74; 44.63)	0.99
	Between day 2 and day 5	-5.16 (-38.6; 20.91)	-18.16 (-32.16; 35.31)	0.87
	Between baseline and day 5	-18.82 (-37.26; 26.58)	-17.96 (-37.63; 68.05)	0.76
	Between day 5 and day 12	-22.67 (-37.79; 34.61)	-10.85 (-40.56; 20.97)	0.91
	Between baseline and day 12	-22.52 (-64.57; 34.82)	-11.06 (-38.53; 37.62)	0.82
% Δ IGF	Between baseline and day 2	-5.91 (-22.51; 11.13)	-5.49 (-18.16; 0.63)	0.76
	Between day 2 and day 5	5.93 (-33.11; 22.98)	8.03 (-7.23; 19.07)	0.81
	Between baseline and day 5	20.69 (-29.48;-10.74)	-7.67 (-15.10; 1.77)	0.84
	Between day 5 and day 12	12.71 (1.61; 30.77)	6.25 (-7.48; 17.65)	0.42
	Between baseline and day 12	-11.23 (-18.74; -4.03)	-0.74 (-10.48; 5.30)	0.56
% Δ TNFα	Between baseline and day 2	-8.86 (-84.48; -43.26)	-22.11 (-56.65; 53.87)^λ^	0.60
	Between day 2 and day 5	-14,47 (-47.72; 13.89)	17.4 (-23.71; 44.31)	0.05*
	Between baseline and day 5	-8.80 (-8.20; 4.76)	-18.99 (-62.45; -5.98)	0.89
	Between day 5 and day 12	3.45 (-26.04; 25.32)	-18.08 (-54.36; 19.49)	0.39
	Between baseline and day 12	-13.63 (-48.23; 20.32)	-43.06 (-76.41; -5.50) ^λ^	0.50
% Δ IL-1β	Between baseline and day 2	-33.58 (-78.25; 7.99)	-5.09 (-17.09; 0.27)	0.04*
	Between day 2 and day 5	12.79 (-29.38; 66.07)	0.31 (-18.25; 34.96)	0.99
	Between baseline and day 5	-18.48 (-69.34; -50.69)	-14.01 (-34.10; 15.73)	0.83
	Between day 5 and day 12	-10.23 (-51.78; 16.73)	-29.47 (-54.06; 16.41)	0.67
	Between baseline and day 12	-1.65 (-65.42; 36.03)	-38.01 (-61.63; 10.98)	0.60
% Δ IL-2	Between baseline and day 2	-13.74 (-21.43; -4.93)	-4.44 (-22.49; 11.48)	0.27
	Between day 2 and day 5	0.98 (-18.08; 7.64)	-4.51 (-15.86; 21.11)	0.76
	Between baseline and day 5	-17.13 (-30.14; -2.05)	-14.15 (-28.51; 10.37)	0.14
	Between day 5 and day 12	3.47 (-10.81; 11.23)	9.84 (-12.26; 27.84)	0.65
	Between baseline and day 12	-0.30 (-19.23; -8.65)	-1.16 (-30.41; 11.45)	0.62
% Δ IL-4	Between baseline and day 2	-18.28 (-61.71; 27.13)	-9.83 (-24.83; -10.37)	0.70
	Between day 2 and day 5	8.17 (-21.17; 94.92)	-4.24 (-19.55; 42.87)	0.76
	Between baseline and day 5	-11.31 (-44.62; 18.36)	-4.33 (-29.21; 44.82)	0.73
	Between day 5 and day 12	-2.44 (-39.26; 17.89)	-14.52 (-41.35; 18.41)	0.61
	Between baseline and day 12	-25.95 (-61.53; 14.40)	-6.86 (-40.83; 11.00)	0.51
% Δ IL-6	Between baseline and day 2	5.45 (-34.19; 168.82)	17.96 (-42.96; 53.75)	0.92
	Between day 2 and day 5	8.91 (-65.54; 105.9)	-50.52 (-73.57; 14.81)	0.22
	Between baseline and day 5	8.60 (-58.10; 44.99)	15.64 (-67.76; 49.44)	0.36
	Between day 5 and day 12	-5.28 (-57.01; 19.53)	2.71 (-81.81; 122.83)	0.82
	Between baseline and day 12	1.56 (-53.79; 21.22)	-35.65 (-91.14; 37.93)	0.12
% Δ IL-8	Between baseline and day 2	-15.29 (-57.69; 8.22)	-26.61 (-59.32; 31.32)	0.42
	Between day 2 and day 5	-42.05 (-71.01; 12.75)	38.34 (-38.54; 65.69)	0.06
	Between baseline and day 5	-40.72 (-87.46; 22.15)	-49.76 (-82.01; 28.01)	0.94
	Between day 5 and day 12	-31.54 (-63.91; 1.88)	-0.48 (-82.67; 23.48)	0.01*
	Between baseline and day 12	-33.33 (-47.41; 1.70)	-75.29 (-89.48; -31.92)	0.08
% Δ IL-10	Between baseline and day 2	27.34 (-6.31; 35.22)	11.22 (-28.71; 40.14)	0.55
	Between day 2 and day 5	7.29 (-18.88; 54.62)	3.26 (-40.33; 23.57)	0.32
	Between baseline and day 5	8.37 (-7.40; 18.48)	-0.14 (-12.11; 27.01)	0.39
	Between day 5 and day 12	-6.38 (-18.93; 7.63)	24.77 (-18.83; 31.62)	0.14
	Between baseline and day 12	-11.21 (-24.76; 7.03)	10.82 (-32.57; 64.22)	0.28

*—р ≤ 0.05 for intergroup comparisons of changes (%) in IL-1β from baseline to day 2; TNFα for days 2–5; and IL-8 for days 5–12;

^£^- р ≤ 0.05 for intragroup dynamics of changes (%) in HGF baseline to day 5 in group 1 and 2;

^γ^—р ≤ 0.05 for intragroup HGF baseline to day 12 in group 1 and 2;

^λ^—р ≤ 0.05 for intragroup dynamics of changes (%) in TNFα baseline to day 5 and TNFα baseline to day 12 in group 2. % Δ: percentage of concentration change

Analysis of dynamic changes in the serum levels of cytokines and growth factors demonstrated that group 1 had more pronounced decreases in the proinflammatory cytokines (IL-1β, TNFα, and IL-8) after ABMMC transplantation compared with group 2.

**Fig 3:**
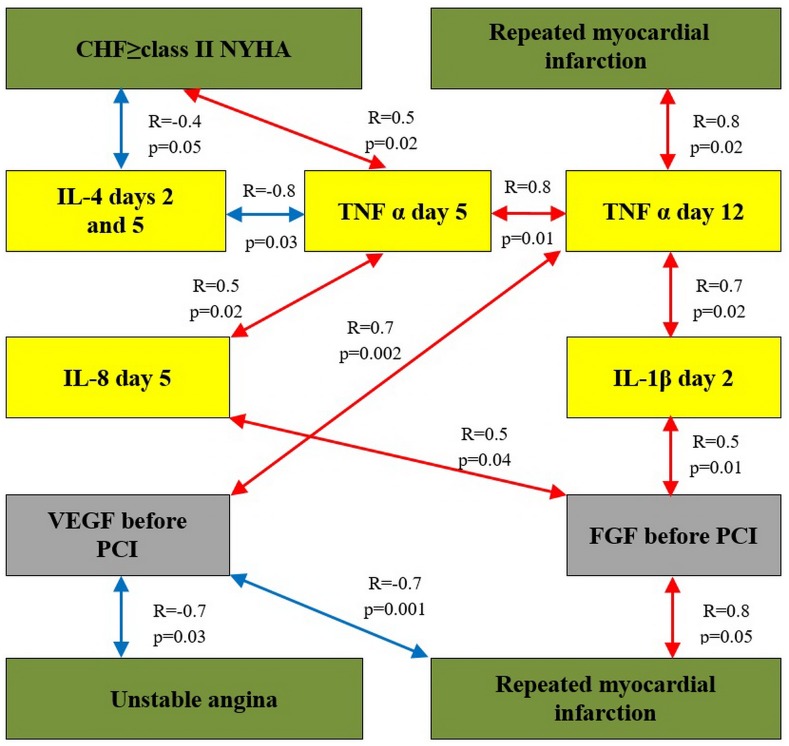
The results of the correlation analysis in group 1. Note: Fig. 3 shows the presence of statistically significant relationships between the absolute serum levels of cytokines and growth factors and the end points as well as between the percentages of their changes at the different times and the end points. Correlation coefficients are placed over the arrows.

**Fig 4:**
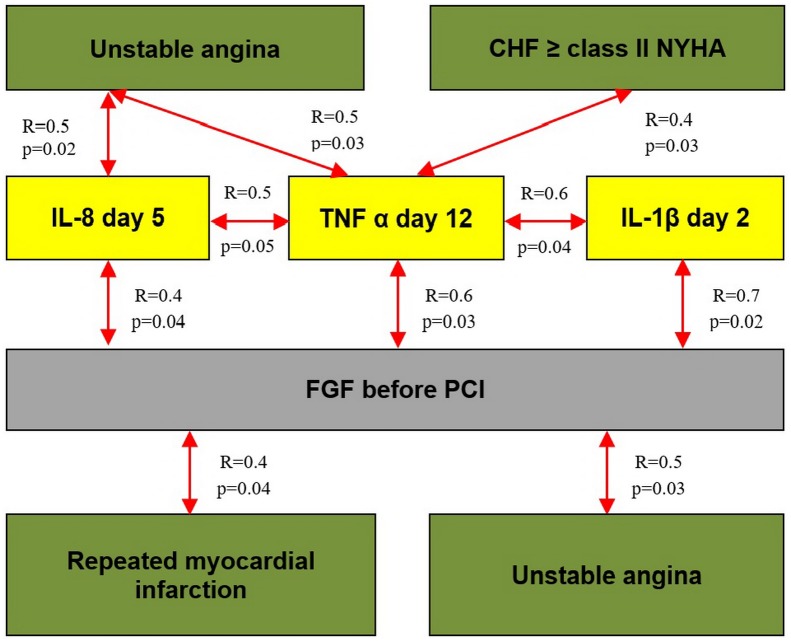
Results of the correlation analysis in group 2. Note: the statistically significant correlations of the cytokines and growth factors with the occurrences of endpoints in group 2 are shown in Fig 4. Correlation coefficients are placed over the arrows.

A correlation analysis was conducted to elucidate the associations between the growth factors and cytokines. The analysis covered those cytokines whose levels significantly changed after PCI (IL-1β and IL-4 at day 2; IL-4 and IL-8 at day 5; and TNFα at days 5 and 12 after PCI). After that, we analyzed the impact of the levels of cytokines and growth factors on the outcomes. Statistically significant correlations were found between the levels of studied growth factors and cytokines (Table 6) as well as between the occurrences of endpoints in each group during the entire period of observation (Figs 3 and 4).

**Table 6:** Correlations between the levels of studied growth factors and cytokines.

Growth factors and cytokines	Group 1 (n = 21)								Group 2 (n = 30)							
	FGF baseline levels		IGF baseline levels		VEGF baseline levels		HGF baseline levels		FGF baseline levels		VEGF baseline levels		HGF baseline levels		IGF baseline levels	
	R	p	R	p	R	p	R	p	R	p	R	p	R	p	R	p
TNF day 5	-0.22	0.33	***0*.*49***	*0*.03*	0.25	0.30	0.21	0.53	0.35	0.08	-0.22	0.32	0.25	0.20	0.09	0.66
TNF day 12	***-0*.*43***	*0*.05*	0.29	0.24	***0*.*67***	*0*.002*	0.13	0.65	***0*.*64***	*0*.03*	0.09	0.70	0.27	0.23	-0.14	0.51
IL1β day 2	***0*.*54***	*0*.01*	0.10	0.67	0.34	0.16	-0.17	0.43	***0*.*70***	*0*.02*	-0.06	0.79	0.07	0.73	0.36	0.08
IL4 day 2	-0.03	0.08	-0.09	0.70	0.02	0.91	0.14	0.60	0.20	0.37	0.05	0.84	0.29	0.20	-0.15	0.51
IL4 day 5	-0.08	0.73	0.21	0.38	0.14	0.55	-0.09	0.72	0.33	0.13	-0.30	0.21	0.27	0.23	-0.32	0.13
IL8 day 5	***0*.*54***	*0*.04*	-0.27	0.24	0.01	0.95	0.40	0.11	***0*.*43***	*0*.04*	0.43	0.04	0.12	0.59	0.19	0.36

*p <0.05—statistically significant differences.

**Fig 5:**
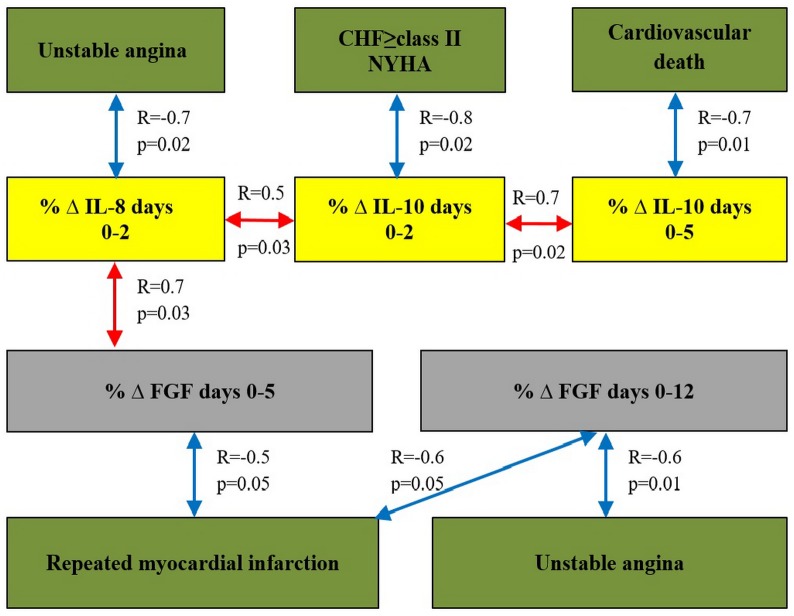
The results of the correlation analysis in group 1. Note: Fig 5 presents statistically significant relationships of the percentages of changes at different times with the end points in group 1. Correlation coefficients are placed over the arrows. Day “0” corresponds to “baseline”.

**Fig 6:**
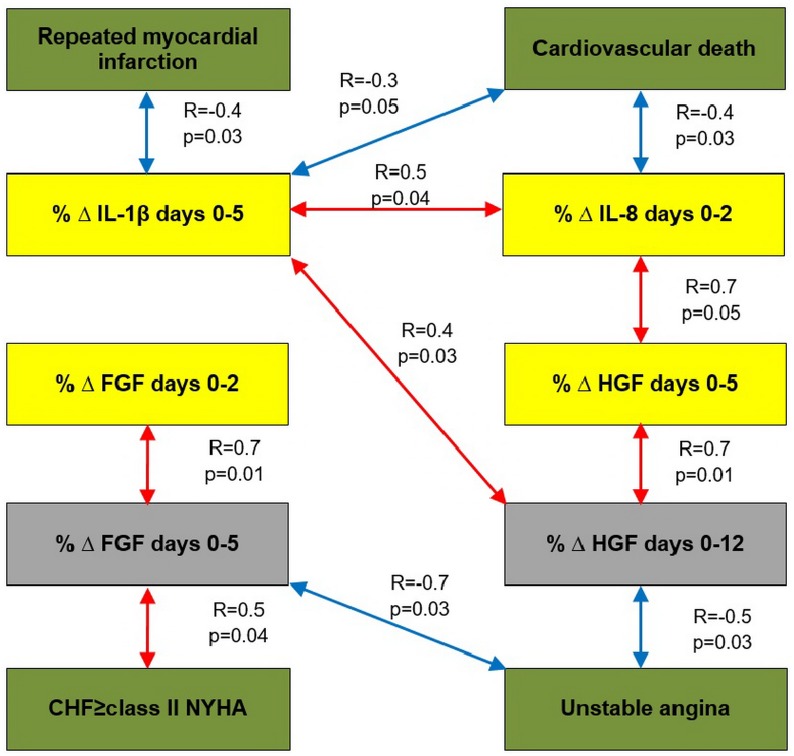
The results of the correlation analysis in group 2. Note: Fig 6 presents statistically significant relationships of the percentages of changes at different times with the end points in group 2. Correlation coefficients are placed over the arrows. Day «0» corresponds to «baseline».

Correlations of the dynamic changes in the concentrations of angiogenic growth factors and cytokines with the end points are presented in Figs 5 and 6.

Positive correlation between the rate of RMI and the level of TNFα at day 12 after PCI (R = 0.8; p = 0.02) and direct correlation between the level of TNFα at day 5 and the rate of CHF class II and more (R = 0.5; p = 0.02) were found in group 1. In control group, the TNFα level at day 12 and the rate of CHF class II and higher (R = 0.4; p = 0.03) directly correlated with the development of unstable angina (R = 0.5; p = 0.03).

In addition, statistically significant positive correlation was found between high concentration of IL-8 at day 5 after PCI and the rate of unstable angina that developed during the follow-up period (7.96 ± 0.96 years) after primary STEMI (R = 0.5; p = 0.02) in control group. Change (%Δ) in IL-8 at days 1–12 after ABMMC transplantation negatively correlated with the development of unstable angina in group 1. In control group, there was a reverse correlation between %Δ IL-8 at days 1–2 and the development of cardiovascular death. This observation confirms the adverse effect of excessive IL-8 production on the course of CAD.

In group 1, negative correlations were observed between %Δ IL-10 at days 0–5 with the cardiovascular mortality (R = -0.7; p = 0.01) and between %Δ IL-10 at days 0–5 and the development of CHF FC II and higher (R = -0.8; p = 0.02).

Positive correlations were observed between the FGF levels and the development of RMI in ABMMC transplantation group (R = 0.8; p = 0.05) and in control group (R = 0.4; p = 0.04); the FGF level correlated with a diagnosis of unstable angina in control group (R = 0.5; p = 0.03).

Decreases in the FGF levels occurred in both groups after the invasive interventions. Taking this into account this, we considered the observed correlation relationships for these end points and time frame consistent (Figs 5 and 6).

Patients of both groups were divided into subgroups depending on the development of the outcomes. High frequency of RMI was observed in patients with significantly higher baseline FGF levels (21.8 pg/mL vs. 16.4 pg/mL; p = 0.02). Thus, higher baseline FGF levels (≥17.2 pg/mL) were associated with adverse long-term prognosis: the development of RMI in both groups.

We also found a negative correlation between the VEGF level before PCI and the rates of repeated AMI (R = -0.7; p = 0.001) and unstable angina (R = -0.7; p = 0.03) in group 1. Percent change in HGF at days 0–12 and the development of unstable angina directly correlated in group 2 (R = -0.5; p = 0.03).

To elucidate predictors of cardiovascular death among different cytokines and growth factors, we used the logistic regression method and built the ROC curves.

**Fig 7:**
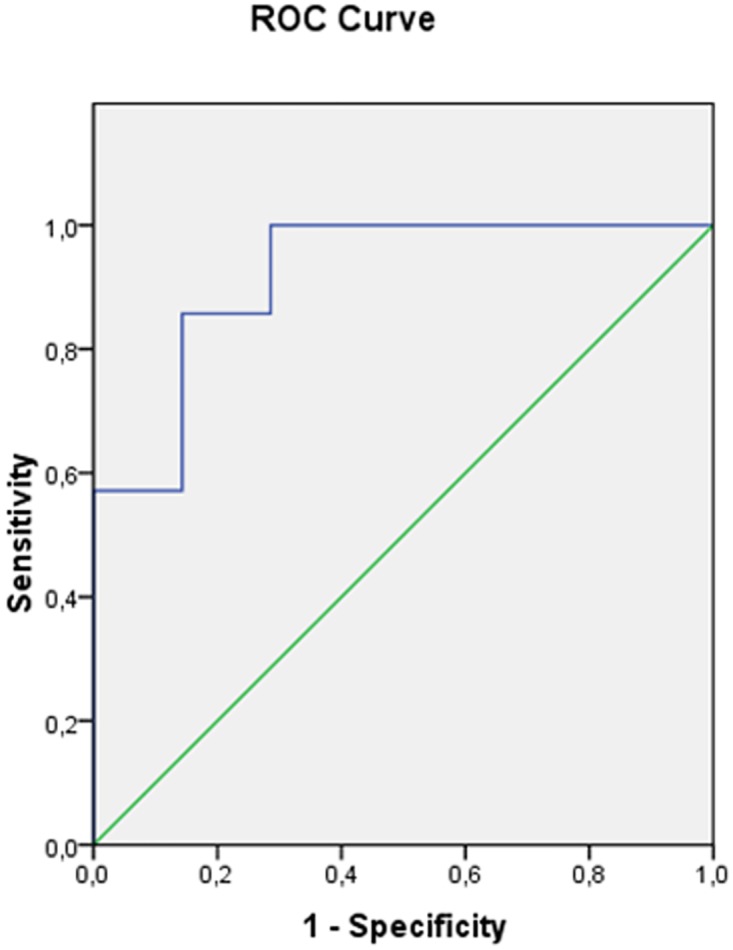
ROC Curve for the IL-10 in group 1.

In group of ABMMC transplantation, predictor of death was the pre-intervention serum level of IL-10 lower than 3.16 pmol/mL. According to data of logistic regression analysis, area under ROC-curve was 0.918 (CI: 0.851–0.948), p = 0.009. Results are presented at Fig 7.

According to logistic regression analysis, other parameters were non-informative.

In addition, we conducted a mathematical analysis to assess the prognostic values of the levels of angiogenic growth factors, proinflammatory cytokines, and anti-inflammatory cytokines in relation to the long-term survival of patients. The serum levels of cytokines and angiogenic growth factors prior to PCI were analyzed. The most informative values were identified with respect to long-term patient prognosis after STEMI. The minimum error probability was detected for VEGF and FGF combination and was 25%. Adding other the angiogenic growth factors and cytokines increased the errors.

## Discussion

According to the results of previously published studies, ABMMC transplantation is associated with a decrease in the mortality and morbidity rates over long-term periods (median follow-up of 5 years) [4, 16]. However, our study did not show positive effects of ABMMC transplantation on the long-term survival of our patients. Meanwhile, the long-term mortality rates in groups 1 and 2 corresponded to the natural course of the disease in such patients and this observation agreed with previously published data [17, 18]. However, the clinical course of CAD among patients who underwent re-examination was more favorable in group 1. Indeed, clinically significant CHF was detected less frequently among patients of this group.

In our study, we analyzed other possible causes of low clinical efficacy of ABMMC transplantation. According to meta-analysis published in 2012, the best long-term clinical results were obtained in case of ABMMC transplantation performed within 7 days after AMI with the amount of ABMMC of more than 10^8^ in patients with left ventricular dysfunction [2]. In our study, the infused amount of ABMMC (120.5 ± 49.2 * 10^6^) was sufficient, but transplantations were performed in patients with preserved LVEF at a later time (at day 20 ± 10 after onset of STEMI). In the long-term, CHF NYHA class II and higher occurred more often among patients who underwent transplantation later than 20 days after STEMI onset. Earlier, we reported that only 3.2% of radionuclide-labeled ABMMC were present in the heart 24 h after transplantation. This fact may also explain the modest results. Repeated administration of ABMMC or introduction of a specific subset of cells may be more beneficial for the improvement of life expectancy and CHF prevention [11, 13].

The main objective of our study was to examine the relationships of the serum levels of growth factors, pro inflammatory cytokines, and anti-inflammatory cytokines with the long-term clinical outcomes in STEMI patients at different times after PCI and ABMMC transplantation. Earlier studies demonstrated macrophages and T-lymphocytes are activated during AMI; nonspecific inflammation develops in the area of myocardial injury. These processes lead to a sharp increase in the synthesis of proinflammatory cytokines. There are two phases in the release of proinflammatory mediators in response to ischemic myocardial injury. IL-1β and TNFα are considered the most important mediators of the first wave that triggers the second phase of the cytokine response consisting in the release of IL-6 and IL-8 [19]. IL-1, IL-6, and TNFα induce biosynthesis of one of the key regulatory cytokines in the immune system: IL-2 which, in turn, affects biosynthesis of early proinflammatory cytokines [20]. To counterbalance the effects of proinflammatory cytokines, IL-4 and IL-10 exerting anti-inflammatory activity are produced. This is the step-by-step way of immune response development in AMI [21]. The observed correlation relationships between proinflammatory and proinflammatory cytokines suggest stage-wise pattern of immunoinflammatory reactions in STEMI.

Moreover, the positive correlation of TNFα with the different endpoints in both groups implies the presence of the adverse effects of an elevated concentration of TNFα after PCI on the clinical course of CAD. It is known that TNFα is expressed soon after the ischemic damage of the cardiomyocytes. This cytokine affects cardiomyocyte survival and apoptosis, mediates inflammatory response, and promotes activation of the proinflammatory cytokines. Earlier studies showed a direct correlation between the level of TNFα and the high frequency of cardiovascular events. TNFα can trigger proinflammatory cytokine synthesis cascade and can modulate secretion of IL-1β, cellular adhesion molecule expression in the endothelial cells, leukocyte activation, and regulation of apoptosis [22, 23]. Our study demonstrated a decrease in the TNFα levels starting from day 2 after ABMMC transplantation. Lower frequencies of clinically significant heart failure and unstable angina in study group were probably due to the paracrine effects of ABMMC.

Studying the changes in concentrations of IL-10 showed that, after ABMMC transplantation, the IL-10 level increased though there were no significant changes between the groups. This marker undoubtedly characterizes severity of the inflammatory response before invasive intervention; IL-10 increment is compensatory in response to excessive production of the proinflammatory cytokines. The effects of IL-10 result in decreased synthesis of these substances. IL-10 suppresses the expression of tissue factor causing hypocoagulation and increasing the release of plasminogen activator [24].

In our study, logistic regression analysis showed that, in group of ABMMC transplantation, low serum level of IL-10, determined before transplantation, was the predictor of death within 7.96 ± 0.96 years after AMI. Negative correlations of the increments in the concentration of this cytokine at day 2 and 5 after ABMMC transplantation with the development of CHF and cardiovascular death were found suggesting the stabilizing effect of IL-10 on CAD course.

It is known that angiogenic growth factors such as HGF [25], IGF [26, 27], FGF [5, 28–32], and VEGF [6–8] are involved in the neovascularization in pathologies; particularly, these molecules exert angiogenic activity in ischemic injury zones in experimental AMI. During recent years, a number of experimental studies suggested that treatment with angiogenic growth factors may promote the development of collaterals to the ischemic tissue in the models of progressive coronary occlusion and AMI [5–7, 25–30]. Angiogenic growth factors reduce infarct size and improve LVEF in animal models of AMI [8, 31, 32]. According to the results of our study, low VEGF levels in patients with STEMI were associated with the occurrence of RMI and unstable angina. It implies that the pre-transplantation serum levels of angiogenic stimulator, VEGF, were associated with the long-term clinical effectiveness of the procedure.

According to the results of experimental animal studies, FGF stimulates angiogenesis [32] and wound healing [33]. Baseline FGF level exerts powerful NO-dependent vasodilatory [34] and myoprotective [35] effects.

Interestingly, we found a correlation between the level of FGF and the number of RMI. Increased serum FGF levels most likely lead to excessive proliferation of the fibroblasts in the atherosclerotic plaques and the myocardium. It can also aggravate CAD course and contribute to adverse postinfarction remodeling of the heart.

Our results agree with data of experimental studies showing that increased production of pro-fibrogenic factors, in particular, FGF, leads to hyperproliferation of the fibroblasts, increased synthesis of collagen and, as a consequence, to tissue fibrosis [5].

In the present study, based on the mathematical analysis, we demonstrated that the VEGF and FGF levels, determined before PCI, were the most informative for prediction of long-term survival of patients after STEMI.

We initially hypothesized that ABMMC could change the serum levels of growth factors, thereby contributing to the vasculogenesis. However, ABMMC administration did not significantly affect these parameters.

## Conclusions

Transplantation of ABMMC modulates the cytokine response in primary STEMI and decreases content of proinflammatory cytokines (TNF-α, IL-1β, and IL-8) in the peripheral blood in subacute period of the disease after PCI. However, these changes are unable to improve the long-term clinical results of the treatment.

The serum levels of FGF, VEGF, and IL-10, determined before PCI and ABMMC transplantation, are prognostically significant indicators of the unfavorable course of CAD after STEMI long-term.

Understanding the role and the mechanisms basis of angiogenic growth factors and cytokines in STEMI is still in its infancy. Additional studies in this field are required.

### Study limitations

The present study was conducted as a single-center study. An unavoidable limitation of the present study lies in the small sample size of our study population. In most patients, PCI was delayed and was performed at day 13±9 after STEMI. However, all patients underwent thrombolytic therapy. Reperfusion of IRCA was achieved at 6.7 ± 1.5 h. Therefore, ABMMC transplantation was performed at a later date, and it was unable to improve the long-term clinical outcomes.

## Supporting information

S1 File CONSORT 2010 checklist. (DOC)

S1 Dataset Raw data of patients enrolled in the study. (XLS)

S1 Protocol Protocol of clinical trial. (DOC)

S2 Protocol Protocol of clinical trial in Russian language. (DOC)
