# Supplementary material for: Relationships of growth factors, proinflammatory cytokines, and anti-inflammatory cytokines with long-term clinical results of autologous bone marrow mononuclear cell transplantation in STEMI
Source: PLoS One. 2017 May 30;12(5):e0176900. doi: 10.1371/journal.pone.0176900 (PMC5448725; doi:10.1371/journal.pone.0176900)
Supplement: S1 Protocol — (DOC) [file pone.0176900.s003.doc]

**CLINICAL PROTOCOL**

The study of the clinical efficacy and safety of transplantation of autologous mononuclear and CD 133^+^ bone marrow cells in acute myocardial infarction-segment elevation ST, and study on the effect of angiogenic growth factors, proinflammatory cytokines in the long-term clinical results of transplantation of autologous mononuclear and CD 133^+^ cells bone marrow

**Version 1 of November 20, 2003**

**STUDY TITLE:**

Clinical efficacy and safety of transplantation of autologous mononuclear and CD 133^+^ bone marrow cells in acute ST-segment elevation myocardial infarction, effects of angiogenic growth factors and proinflammatory cytokines in the long-term clinical results of autologous mononuclear and CD 133^+^ bone marrow cell transplantation.

**Organizer of the study:**

Federal State Budgetary Scientific Institution “Research Institute for Cardiology”, Tomsk, Russian Federation

The sponsor: Russian Academy of Sciences

**RESEARCH TEAM**

**Director of Research Team:**

**Karpov, Rostislav S.**

M.D., Ph.D., Doctor of Medical Sciences, Professor, Member of the Russian Academy of Sciences, Head of Therapy Department of the Siberian Sate Medical University, Director of RI Cardiology.

Address: 111a Kievskaya Street, Tomsk, 634012

Phone (work): +7 (3822) 55-34-81

E-mail: tvk@cardio.tsu.ru

**Markov, Valentin A.**

M.D., Ph.D., Doctor of Medical Sciences, Professor, Head of Cardiology Department at the Continuous Medical Education Faculty of the Siberian Sate Medical University, Head of Cardiac Emergency Department, RI Cardiology.

Address: 111a Kievskaya Street, Tomsk, 634012

Phone (work): +7 (3822) 49-32-06

E-mail: markov@cardio-tomsk.ru

E-mail: [markov@cardio-tomsk.ru](https://docviewer.yandex.ru/r.xml?sk=07d542916b67de5e3f6a93fcf5fd9587&url=mailto%3Amarkov@cardio-tomsk.ru)

**Ryabov, Vyacheslav V.**

M.D., Ph.D., Doctor of Medical Sciences, Leading Research Fellow of Cardiac Emergency Department, RI Cardiology; Docent of Cardiology Department at the Continuous Medical Education Faculty of the Siberian Sate Medical University.

Address: 111a Kievskaya Street, Tomsk, 634012

Phone (work): +7 (3822) 55-83-60

E-mail: [rvvt@cardio-tomsk.ru](mailto:rvvt@cardio-tomsk.ru)

**АIM:** to investigate the long-term results of transplantation of the autologous bone marrow mononuclear cells and CD133^+^ bone marrow cells in patients (pts) with primary STEMI, to determine the serum levels of growth factors and cytokines in patients with STEMI, to detect structural and functional changes of the heart in patients during and late after first-time acute myocardial infarction, and to determine the impact of cell transplantation on long-term clinical outcomes.

**BACKGROUND**

Acute myocardial infarction (AMI) is often considered one of the main causes of heart failure. Major factors contributing to chronic heart failure (CHF) after AMI include extensive myocardial infarction (MI) and state of coronaries. Death of cardiomyocytes, arterioles, and capillaries in the myocardial infarction area is irreversible and subsequently results in the formation of scar tissue (Orlic D., Kajstura J., et al., 2001). Following remodeling of the left ventricle (LV) leads to progressive dilatation and disruption of its geometry which represents morphological substrate of CHF (Pfeffer MA, Brunwald E., 1990).

During recent years, immune system responses in the presence of myocardial infarction and CHF have been extensively studied. Inflammatory responses and production of cytokine are significantly augmented in AMI leading to remodeling of the heart and following onset of CHF (Nian M., Lee P. et al., 2003). In this regard, primary tasks of modern cardiology consist in recanalization of the infarct-related coronary artery (IRA) and restoration of the myocardial perfusion in patients with AMI. Implementation of various methods of emergency myocardial reperfusion decreased early in-hospital lethality and improved long-term prognosis in these patients. However, CHF, caused by AMI, remains relevant clinical problem.

Transplantation of autologous stem cells is a novel trend in treatment of AMI and prevention of LV remodeling. Prospects for recovery of the myocardium via transplantation of the stem cell with various phenotypes are currently under investigation. Promising results have been generated in various experimental models of ischemic and non-ischemic heart diseases where beneficial effects of transplanted cells were documented. The beneficial effects were mediated by the involvement of the transplanted cells into the myocardial contraction, improvement of mechanical properties of the heart, and paracrine effects of transplantation consisting in the induction of neoangiogenesis (Kocher A.A. et al., 2001, Li R.-K. et al., 1996; Orlic D. et al., 2001; Zhang S. et al., 2003). In these studies, researchers used the following cells for transplantation: embryonic stem cells (ESC), bone marrow stem cells (BMSC), and skeletal myoblasts. Feasibility of cell survival after the transplantation, cell integration into the myocardium of a recipient, and improvement of the cardiac function was confirmed (Сhierchia S., Deferrari L., 2003). Experimental studies on animals showed that bone marrow mononuclear cells (BMMC) can both trigger regeneration of the myocardium in the infarction zone and to trigger myogenesis and angiogenesis with the following improvement of cardiac function (Orlic D., Kajstura J. et al., 2003). The first pilot clinical trials studying the efficacy of BMMC transplantation have been already performed (Hamano K. et al 2001; Strauer B.E., Kornowski R., 2003; Shumyakov V.I. et al.m 2003). However, all these studies were performed at the preclinical stage; the indications and contradictions for administration of this treatment modality have not been unidentified yet. The question regarding optimal time frame for administration of cell transplantation remains unsolved. It is also unclear which type of cells is preferable for the transplantation: ESC, autologous BMMC, CD133^+^, pure pool of stem cells or the cells that underwent culturing and reprograming. Available literature does not present data on long-term clinical results of cell transplantation in AMI.

The safety and efficacy of autologous bone marrow cell transplantation as well as mechanistic basis of cell transplantation and its effects on cytokines and growth factors in patients with AMI require further studies.

**SUBJECT POPULATION**

The subject population comprises men and women age 18 years and older with STEMI. Each potential subject will have to meet the inclusion criteria and should not be exclusion criteria.

**INCLUSION CRITERIA**

1.​ Age 18 to 75 years,

2.​ Primary STEMI

3.​ Reperfusion time of the infarct-related coronary artery (IRCA) more than 4 h

4.​ Admission at the Coronary Care Unit during the 24 hours after onset of STEMI

**EXCLUSION CRITERIA:**

1.​ Atrial fibrillation, a permanent form

2.​ Valvular heart disease

3.​ Severe comorbidity

4.​ The refusal of the patient to conduct the necessary research

**STUDY DESING**

Single-center, prospective, open, randomized, parallel controlled study.

Planned number of included patients is 90 patients.
Planned date of the inclusion of the first patient is Dec. 1, 2003.
Planned date of the inclusion of the last patient is 20 December 2006.
Planned date of completion of the study is 1 February 2015.
Monitoring of patients will continue until 8 years after suffering a first myocardial infarction.
Basic data from the medical records will be recorded in an individual protocol.

Pts will be randomized to three groups by envelopes: group 1 - pts who will underwent percutaneous coronary intervention (PCI) and ABMMC transplantation; group 2 - pts who will underwent PCI and transplantation of autologous CD133 + bone marrow cells, group 3 - will be comprised pts with only PCI.

**STUDY PROCEDURES**

Patients meeting inclusion and exclusion criteria will be invited to participate in the study. Patient’s consent will be confirmed by signing the written consent form.

Randomization procedure will be conducted by the envelope method after obtaining the written confirmation of patient's consent to participate in the study.

Puncture of the anterior superior iliac spine under local anesthesia will be conducted in patients of groups 1 and 2 4–5 hours before the transplantation of ABMMC or autologous CD133^+^ bone marrow cells. Bone marrow aspirate (100 mL) will be acquired into two 60-mL syringes containing 10 mL of sterile saline and 25000 IU of heparin. After that, ABMMC and autologous CD133^+^ bone marrow cells will be isolated by the method of gradient centrifugation (density gradient Histopaque-1077) and separated from erythrocytes, thrombocytes, and granulocytes. We plan to allocate 5–10 • 10^6^ CD133+ BMC and 5-10 • 10^6^ ABMMC. Cellular phenotyping will be performed by the method of flow cytofluorometry (FACSCalibur, Biosciences, USA).

Magnetic labeling with magnetic microgranules CD133 MicroBead (Miltenyi Biotec GmbH, Germany) will be carried out to isolate CD133^+^ BMC. CD133^+^ progenitor cells labeled using a hapten-conjugated monoclonal primary antibody and anti-hapten antibody bound to MACS MicroBead microgranules. Positive magnetic separation will be conducted in the separation column in a magnetic field with the device MidiMACS. The purity of cell populations and their viability will be assessed by flow cytofluorometry after immunofluorescent staining with specific CD133/2 (AC141)-PE dye and vital dye 7-AAD by using BD FACSCalibur device (USAVital staining of ABMMC with trypan blue will be used to assess cell viability.

Suspension of 2-4 • 10^6^ ABMMC in 1-mL of heparinized solution (20 U of heparin in 1 mL) will be prepared for transplantation. Intracoronary infusion of ABMMC will be performed by the method of passive passage to the IRA at a rate of 4–8 mL/min. Distribution of ^99m^Tc-HMPAO-labeled mononuclear cells in patient’s body will be studied by scintigraphy (Nuclear Gamma Camera 500, Technicare, USE-Germany).

Plasma samples will be collected to determine serum levels of proinflammatory cytokines and growth factors: tumor necrosis factor α (TNFα), interleukin-1β (IL-1β), hepatocyte growth factor (HGF), vascular endothelial growth factor (VEGF), fibroblast growth factors (FGF), insulin-like growth factors (IGF), and transforming growth factor (TGF) before PCI and 2, 5, and 12 days after the procedure. The levels of cytokines and growth factors will be assessed by enzyme-linked immunosorbent assay: HGF by Biosource (Belgium), VEGF by Cytimmune (USA), FGF by Biosource (Belgium), IGF by DSL (USA), TGF by Biosource (Belgium), IL-1β by Protein contour (Russia), and TNFα by Protein contour (Russia).

**The following clinical parameters will be determined at a time of discharge from a hospital:**

1. Six-minute walk test will be performed to assess the functional class of heart failure. The results will be evaluated according to the criteria of functional classes of the New York Heart Association (NYHA).

2. Functional class of angina will be evaluated according to the classification of the Canadian Cardiovascular Society.

3. The size of myocardial infarction will be determined by counting the index QRS in 12 standard ECG leads by Selvester code to modify Wagner GS et al. (1982).

4. Ultrasound examination of the heart will be held on the ultrasound system VIVID 7, GE Vingmed Ultrasound, Norway.

Patients, enrolled in the study, will be interviewed over the phone 6 months, 12 months, and 8 years after the procedure and will be invited to follow-up visits.

During follow-up visits, we will evaluate the clinical status of patients (the presence of angina, stage and functional class of heart failure, compliance, and quality of life) and the clinical course of cardiovascular disease (complications, the incidence of cardiovascular events, death).

**The following clinical parameters will be determined at follow-up visits:**

1. Quality of life will be assessed using the Minnesota Quality of Life Questionnaire in patients with CHF.

2. Six-minute walk test will be performed to assess the functional class of heart failure. The results will be evaluated according to the criteria of functional classes of the New York Heart Association (NYHA).

3. Assessment of signs of coronary insufficiency will be defined by the presence of stable angina, recurrent myocardial infarction, and unstable angina after myocardial infarction. Evaluation of the functional class of angina will be carried out according to the classification of the Canadian Cardiovascular Society.

4. History of arrhythmias and cardiac conduction defects developed after AMI will be documented.

5. Patient adherence to standard drug therapy will be investigated by the Moriscos-Green test.

**The following laboratory and instrumental methods of investigation will be carried out during the follow-up visits:**

1. Ultrasound examination of the heart will be performed by using the ultrasound system VIVID 7, GE Vingmed Ultrasound, Norway.

2. Coronary ventriculography will be performed if clinically indicated.

3. Serum BNP levels will be determined with the help of the Triage BNP Test analyzer Triage Meter, Biosite, USA.

**The following events are defined as end points:**

1. Death.

2. Repeated myocardial infarction.

3. Unstable angina.

4. Chronic heart failure (CHF) ≥class II (NYHA).

5. Stroke.

Besides, we will assess the number of planned and emergency admissions to a hospital due to cardiovascular diseases with diagnosis of unstable angina and repeated myocardial infarction.

**The following events are defined as safety endpoints:**

1. Clinically significant cardiac arrhythmias including life-threatening arrhythmias.

2. New-onset oncological diseases.
