# Supplementary material for: Relationships of growth factors, proinflammatory cytokines, and anti-inflammatory cytokines with long-term clinical results of autologous bone marrow mononuclear cell transplantation in STEMI
Source: PLoS One. 2017 May 30;12(5):e0176900. doi: 10.1371/journal.pone.0176900 (PMC5448725; doi:10.1371/journal.pone.0176900)
Supplement: S2 Protocol — (DOC) [file pone.0176900.s004.doc]

**ПРОТОКОЛ КЛИНИЧЕСКОГО ИССЛЕДОВАНИЯ**

**Исследование по изучению клинической эффективности и безопасности трансплантации аутологичных мононуклеарных и CD 133 ^+^ клеток костного мозга при остром инфаркте миокарда с подъемом сегмента ST, a также по изучению влияния ангиогенных факторов роста, провоспалительных цитокинов на отдаленные клинические результаты трансплантации аутологичных мононуклеарных и CD 133 ^+^ клеток костного мозга.**

**Версия 1 от 20 ноября 2003**

**НАЗВАНИЕ:**

Исследование по изучению клинической эффективности и безопасности трансплантации аутологичных мононуклеарных и CD 133 ^+^ клеток костного мозга при остром инфаркте миокарда с подъемом сегмента ST, a также по изучению влияния ангиогенных факторов роста и провоспалительных цитокинов на отдаленные клинические результаты трансплантации аутологичных мононуклеарных и CD 133 ^+^ клеток костного мозга.

**ОРГАНИЗАТОР ИССЛЕДОВАНИЯ:**

ФГБУ «НИИ кардиологии» СО РАМН, Томск

Спонсор исследования: Российская академия медицинских наук

**ИССЛЕДОВАТЕЛЬСКАЯ КОМАНДА**

**Руководители исследовательской группы**

**Карпов Ростислав Сергеевич**, докт. мед. наук, профессор, академик РАМН, заведующий кафедрой факультетской терапии с курсом клинической фармакологии ГБОУ ВПО СибГМУ Минздрава России, директор ФГБУ «НИИ кардиологии» СО РАМН.

Адрес: 634012, г. Томск, ул. Киевская, 111а.

E-mail: tvk@cardio.tsu.ru

**Марков Валентин Алексеевич**, докт. мед. наук, профессор, заведующий кафедрой ФПК и ППК кардиологии ГБОУ ВПО СибГМУ России, руководитель отделения неотложной кардиологии ФГБУ «НИИ кардиологии» СО РАМН.

Адрес: 634012, г. Томск, ул. Киевская, 111а.

E-mail: [markov@cardio-tomsk.ru](mailto:markov@cardio-tomsk.ru)

**Рябов Вячеслав Валерьевич**, старший научный сотрудник отделения неотложной кардиологии ФГБУ «НИИ кардиологии» СО РАМН, доцент кафедры кардиологии ФПК и ППС ГБОУ ВПО СибГМУ Минздрава России.

Адрес: 634012, г. Томск, ул. Киевская, 111а.

E-mail: rvvt@cardio-tomsk.ru

**ЦЕЛЬ ИССЛЕДОВАНИЯ**

Изучить отдаленные клинические результаты трансплантации аутологичных мононуклеарных и CD133+клеток костного мозга, а также определить значение факторов роста и цитокинов в развитии структурно-функциональной перестройки сердца у больных во время и в отдаленные сроки после острого первичного инфаркта миокарда и определить их влияние на отдаленные клинические результаты трансплантации аутологичных мононуклеарных и CD 133 + клеток костного мозга.

**ВСТУПЛЕНИЕ**

Как известно, острый инфаркт миокарда (ОИМ) часто является одной из основных причин развития ХСН. Очевидно, что ведущими факторами возникновения ХСН после ОИМ являются обширность инфаркта миокарда (ИМ) и состояние коронарного русла. Гибель кардиомиоцитов, артериол и капилляров в зоне ИМ необратима, что в последующем ведет к формированию рубцовой ткани (Orlic D., Kajstura J. с соавт., 2001). Последующее ремоделирование левого желудочка (ЛЖ), приводящее к прогрессирующей дилатации и нарушению его геометрии, является морфологическим субстратом развития ХСН (Pfeffer M.A., Brunwald E., 1990). В последние годы активно изучается значение реакции иммунной системы при ИМ и ХСН. Воспалительный ответ и образование цитокинов особенно повышены при ОИМ, что также ведет к ремоделированию сердца с последующим развитием ХСН (Nian M., Lee P. с соавт., 2003). В связи с этим, одной из основных задач современной кардиологии в лечении пациентов с ОИМ является реканализация инфаркт-связанной коронарной артерии (ИСКА) и восстановление перфузии миокарда. Несмотря на то, что внедрение в клиническую практику различных методов экстренной реперфузии миокарда привело к уменьшению ранней госпитальной летальности и улучшению отдаленного прогноза у этих пациентов, ХСН вследствие перенесенного ОИМ остается актуальной клинической проблемой.

Трансплантация аутологичных стволовых клеток является новым направлением в лечении ОИМ и профилактике ремоделирования ЛЖ. В настоящее время возможности восстановления миокарда путем трансплантации клеток различных фенотипов в миокард интенсивно изучаются. Обнадеживающие результаты получены в различных экспериментальных моделях ишемической и неишемической болезнях сердца, в которых установлены благотворные эффекты трансплантированных клеток, обусловленные, как участием клеток в сокращении миокарда, улучшением механических свойств сердца, так и паракринными эффектами трансплантации, выражающимися в индукции неоангиогенеза (Kocher A.A. с соавт., 2001; Li R.-K. с соавт., 1996; Orlic D. с соавт., 2001; Zhang S. с соавт., 2003). В проведенных исследованиях для трансплантации использовали эмбриональные стволовые клетки (ЭСК), стволовые клетки костного мозга (СККМ), скелетные миобласты. Подтверждена возможность выживания клеток после трансплантации, интеграции их в миокард реципиента, улучшения функции сердца (Сhierchia S., Deferrari L., 2003). В исследованиях на животных показано, что мононуклеарные клетки костного мозга (МККМ) способны вызывать не только регенерацию зоны ИМ, но и мио-и ангиогененез с последующим улучшением функции сердца (Orlic D., Kajstura J. с соавт., 2003).

К настоящему моменту времени выполнены первые предварительные клинические испытания эффективности трансплантации аутологичных МККМ (Strauer B.E., Kornowski R., 2003; Шумаков В.И, Казаков Э.Н. и соавт., 2003; Hamano K., Nishida M. и соавт., 2001). Тем не менее очевидно, что все исследования, связанные с использованием этого метода лечения, находятся в стадии клинических испытаний, не определены показания и противопоказания к его использованию. Не решен вопрос об оптимальных сроках проведения клеточной трансплантации. Неясно, какой вид клеток предпочтительнее использовать для трансплантации – ЭСК, аутологичные МККМ, CD133^+^ ККМ, чистый пул стволовых клеток или клетки, прошедшие культивирование и перепрограммирование. Отсутствуют данные по отдаленным клиническим результатам клеточной трансплантации при ОИМ.

Таким образом, в настоящее время вопросы, касающиеся безопасности и эффективности трансплантации аутологичных клеток костного мозга у больных ОИМ, механизмы их воздействия на миокард и систему цитокинов и факторов роста остаются открытыми.

**ПОПУЛЯЦИЯ ИССЛЕДОВАНИЯ**

Мужчины и женщины старше 18 лет с острым инфарктом миокарда с подъемом сегмента ST соответствующие критериям включения и не имеющие критериев исключения

**КРИТЕРИИ ВКЛЮЧЕНИЯ:**

- Возраст от 18 до 75 лет
- Первичный инфаркт миокарда с подъемом сегмента ST
- Срок поступления в блок интенсивной терапии в первые 24 часа от начала заболевания
- Время реперфузии инфаркт-связанной коронарной артерии не ранее 4 часов после начала острого первичного трансмурального инфаркта миокарда

**КРИТЕРИИ ИСКЛЮЧЕНИЯ:**

- Мерцательная аритмия, постоянная форма
- Клапанные пороки сердца
- Тяжелая сопутствующая патология
- Отказ пациента от проведения необходимых исследований

**ДИЗАЙН ИССЛЕДОВАНИЯ**

Одноцентровое проспективное, открытое, рандомизированное параллельное контролируемое исследование (рис. 1)

Планируемое количество включенных больных - 90 пациентов.

Планируемая дата включения первого пациента 01 декабря 2003 года.

Планируемая дата включения последнего пациента 20 декабря 2006 года.

Планируемая дата окончания исследования 01 февраля 2015 года.

Наблюдение за пациентами будет продолжаться до истечения 7 лет после перенесенного первичного инфаркта миокарда.

Основные данные из историй болезни будут фиксироваться в индивидуальном протоколе исследования.

Пациенты будут рандомизированы на две группы методом конвертов: 1-я группа – проведено стентирование ИСКА и трансплантация аутологичных мононуклеарных клеток костного мозга (АМКК), 2-я группа ‑ стентирование ИСКА и трансплантация аутологичных CD133^+^ клеток костного мозга (CD133^+^ ККМ), 3-я группа ‑ стентирование ИСКА.

**Рис 1. Дизайн исследования.**

Острый первичный передний крупноочаговый инфаркт миокарда

Рандомизация

Основная группа, n=45

Группа контроля, n=45

Внутрикоронарное введение мононуклеарных аутологичных клеток КМ

Внутрикоронарное ведение аутологичных CD133^+^ клеток КМ

Клинический осмотр

Определение провоспалительных цитокинов, ангиогенных факторов роста

ЭхоКГ

Повторное обследование через 6, 12-мес

Клинический осмотр

ЭхоКГ

Повторное обследование через 7 лет

Клинический осмотр

ЭхоКГ

При наличии показаний проведение КВГ

**ПРОЦЕДУРЫ ИССЛЕДОВАНИЯ**

Всем пациентам, соответствующим критериям включения и исключения будет предложено участие в исследовании. Согласие пациента будет подтверждено письменно.

После получения от пациента письменного подтверждения желания участвовать в исследовании, необходимо проведение процедуры рандомизации методом конвертов.

У пациентов 1-й и 2-й группы за 4-5 часов до процедуры трансплантации АМКК либо CD133^+^ ККМ будет проводиться пункция передне-верхней ости крыла подвздошной кости под местной анестезией для получения аспирата костного мозга в объеме 100 мл в два 60-миллилитровых шприца, содержащих 25000 Ед гепарина и 10 мл стерильного физиологического раствора. Затем методом градиентного центрифугирования (градиент плотности Histopaque-1077) будет выделяться АМКК (у пациентов 1-й группы) либо CD133^+^ ККМ (у пациентов 2-й группы) посредством отделения их от эритроцитов, тромбоцитов, гранулоцитов. Планируется выделение 5-10*10^6^ CD133+ ККМ и 5-10*10^6^ АМККМ. Методом проточной цитофлуометрии будет выполнено фенотипирование трансплантируемых клеток. Для выделения CD133^+^ ККМ будет проводиться магнитное мечение с помощью магнитных микрогранул CD133 MicroBead (Miltenyi Biotec GmbH, Germany). CD133^+^ прогениторные клетки будут метиться с помощью гаптен - конъюгированных первичных моноклональных антител и анти-гаптен антител, связанных MACS MicroBead микрогранулами. Позитивная магнитная сепарация будет проводиться на сепарационных колонках в магнитном поле на приборе MidiMACS. Чистота популяции клеток и их жизнеспособность будет оценена с помощью проточной цитофлуометрии после иммунофлуоресцентного окрашивания специфическим красителем CD133/2 (АС141) -РЕ и витальным красителем 7-AAD на приборе BD FACSCalibur (USA). Для подсчета жизнеспособности АМККМ, они будут окрашены витальным красителем - трипановым синим.

Для трансплантации АМКК будет приготовлена суспензия АМКК 2-4 *10^6^ в 1 мл гепаринизированного раствора (20 Ед гепарина в 1 мл). Введение АМККМ будет проведено интракоронарно методом пассивного пассажа в течение 5 мин после эффективной баллонной ангиопластики и стентирования ИСКА со скоростью 4-8 мл/мин. Распределение АМКК в организме больного будет изучаться методом радионуклидной индикации клеточной взвеси с помощью 40-60 мКи ^99m^Tc-HMPAO-labeled («Сeretec») в соответствии с руководством для метки лейкоцитов корпорации «Nycomed Amersham» непосредственно перед их внутрикоронарным введением. Сцинтиграфическая индикация распределения меченных АМКК в организме пациента будет выполняться в планарном режиме через 30 мин, 2,5 и 24 ч после их введения (Nuclear Gamma Camera 500, Technicare, USE-Germany).

Будет производиться забор крови для определения следующих факторов роста и цитокинов: гепацитарный фактор роста (HGF), васкулоэндотелиальный фактор роста (VEGF), фактор роста фибробластов (FGF), инсулиноподобный фактор роста 1 (IGF1), интерлейкин-1 (IL-1 β), фактор некроза опухоли - α (TNFα), трансформирующий фактор роста (TGF) до проведения ЧТКА и трансплантации стволовых клеток и на 2,5,12 сутки после инвазивных процедур. Определение цитокинов и факторов роста будет выполняться иммуноферментным методом (ELISA-тест) с использованием набора реагентов: HGF – Biosource (Бельгия), VEGF - Cytimmune (США), FGF -  Biosource (Бельгия), IGF - DSL (США), TGF - Biosource  (Бельгия), IL-1 β – «Протеиновый контур», Россия, TNFα - Протеиновый контур».

**На момент выписки из стационара будут определены следующие клинические параметры:**

1. Для оценки функционального класса ХСН будет проводиться тест 6-ти минутной ходьбы, результаты будут оценены согласно критериям функциональных классов Нью-Йоркской ассоциации сердца (NYHA)
2. Оценка функционального класса стенокардии будет проводиться согласно классификации Канадской ассоциации кардиологов
3. Будет определен размер постинфарктного кардиосклероза методом подсчета индекса QRS в 12 стандартных отведениях ЭКГ по Selvester code в модификации Wagner G.S. et al. (1982)
4. Будет проведено ультразвуковое исследование сердца на ультразвуковой системе «VIVID 7, GE Vingmed Ultrasound, Norway»

Включенные в исследование пациенты будут опрошены путем телефонного интервью 6 мес, 12 мес, 7 лет и приглашены на контрольное обследование.

Во время визита будет оцениваться клиническое состояние больных (наличие стенокардии, стадия и функциональный класс ХСН, приверженность стандартной терапии после ОИМ, качество жизни), течение заболевания (осложнения, частота сердечно-сосудистых событий, летальные исходы).

**При контрольном обследовании будут определены следующие клинические параметры:**

1. Качество жизни пациентов будет оцениваться с помощью Миннесотского опросника качества жизни у больных с ХСН.
2. Для оценки функционального класса ХСН будет проводиться тест 6-ти минутной ходьбы, результаты будут оценены согласно критериям функциональных классов Нью-Йоркской ассоциации сердца (NYHA).
3. Оценка признаков коронарной недостаточности будет проведена по наличию стабильной стенокардии, повторных инфарктов миокарда, эпизодов нестабильной стенокардии в отдаленный сроки после ОИМ. Оценка функционального класса стенокардии будет проводиться согласно классификации Канадской ассоциации кардиологов.
4. Наличие в анамнезе нарушений ритма и проводимости сердца, развившиеся после острого инфаркта миокарда.
5. Приверженность пациентов к стандартной медикаментозной терапии будет исследована с помощью теста Мориски-Грина.

**При контрольном осмотре будут выполнены инструментальные и лабораторные методы исследования:**

1. Будет выполнена эхокардиография на ультразвуковой системе «VIVID 7, GE Vingmed Ultrasound, Norway» с целью определения глобальной систолической функции ЛЖ: фракцию выброса ЛЖ, конечный систолический объем, конечный диастолический объем и конечный систолический индекс (КСИ); показатели насосной функции сердца: ударного объема и сердечного выброса ЛЖ. Количественное определение степени нарушения локальной сократимости будет проводиться путем расчета индекса нарушения локальной сократимости.
2. При наличии клинических показаний – коронаровентрикулография.
3. Будет определен сывороточный уровень BNP с помощью панели Triage BNP Test на анализаторе «Triage Meter», Biosite, USA.

**Конечными точками определены следующие события:**

1. Смерть
2. Повторный инфаркт миокарда
3. Нестабильная стенокардия
4. ХСН II и более функционального класс
5. Острое нарушение мозгового кровообращения

Кроме того, будет оценено количество госпитализации по поводу сердечно-сосудистых заболеваний: плановых, а также в порядке скорой медицинской помощи с диагнозом нестабильная стенокардия, повторный инфаркт миокарда.

**Определены следующие показатели безопасности:**

1. Клинически значимые нарушения ритма сердца, включая появление жизнеугрожающих нарушений ритма сердца (желудочковая тахикардия, фибрилляция желудочков).
2. Появление новых онкологических заболеваний.
